# Nuclear factor translocation and acute anterior uveitis

**Published:** 2011-01-18

**Authors:** Jing Wang, Hong Lu, Xiaofeng Hu, Wei Chen, Zhuozai Xu, Shang Li, Yingzhi Xu

**Affiliations:** 1 Department of Ophthalmology, Chaoyang Hospital, Capital Medical University, Beijing, China; 2 Department of Ophthalmology, Haidian Maternal and Child Health Hospital, Tonji Medical University, Beijing, China

## Abstract

**Purpose:**

To investigate the roles of activation of macrophages isolated from C3H/HeN and C3H/HeJ mice and stimulated by lipopolysaccharide (LPS), and toll-like receptor 4-mediated signal transduction in the development of acute anterior uveitis.

**Methods:**

Establish animal models with acute anterior uveitis by intraperitoneal injection of vibrio cholera endotoxin into C3H/HeN mice (wild type) and C3H/HeJ mice (toll-like receptor 4 (*TLR4*) gene defection type). Peritoneal macrophages were obtained from C3H/HeN and C3H/HeJ mice. Immunofluorescence staining was used to identify the F4/80+ positive cells (iris, peritoneal macrophages) and to observe the expression of TLR4, myeloid differentiation factor 88 (MyD88), and nuclear factor kappa B (NF-κB), with or without LPS (1 μg/ml). To investigate the importance of TLR4 in the signal pathway, a group, blocked by anti-TLR4 antibody before LPS stimulation, was added to theC3H/HeN mice sample.

**Results:**

In vitro, in C3H/HeN mice, Iris posterior synechia was found 24 h later. However, an inflammation reaction was not found in the anterior chamber of the C3H/HeJ mice. In cell culture, TLR4 expression was observed in peritoneal macrophages of the C3H/HeN mice, both with and without LPS stimulation. TLR4 was primarily expressed in the membrane and no significant difference in inflorescence intensity (p=0.081) was found among the groups. MyD88 was expressed in the cytoplasm and the nucleus. There is statistical significance in the fluorescence intensity among groups of C3H/HeN mice (p<0.0001). NF-κB was primarily expressed in the cytoplasm before LPS stimulation. However, this occurred 1 h after LPS stimulation and could be observed in the nucleus. Three hours after LPS stimulation, the expression of NF-κB could not be detected in the cytoplasm or the nucleus. The fluorescence intensity of TLR4 and MyD88 expression showed no significant difference (p=0.113) between the anti-TLR4 antibody pretreatment group and the other groups of C3H/HeN mice. However, in the anti-TLR4 antibody pretreatment group, 1 h to 24 h after LPS stimulation, NF-κB only expressed in the cell membrane. Peritoneal macrophages from all groups of C3H/HeJ mice showed no obvious changes in morphology and size after LPS stimulation (p=0.257). TLR4 was primarily expressed in the cell membrane, and fluorescence intensity showed no statistical significance (p=0.228); MyD88 was expressed in the cytoplasm and the nucleus and there was no significant difference in fluorescence intensity among the groups (p=0.108); NF- κB was expressed in the cytoplasm, without LPS stimulation; however, 1 h after LPS stimulation, it appeared in the cell membrane and persisted until 24 h.

**Conclusions:**

Acute anterior uveitis can be induced in wild-type mice, but it cannot be induced in TLR4 gene-deficient mice. The variation of expression of TLR4, and its downstream signal transduction molecules, MyD88 and NF-κB, after LPS stimulation in vitro, suppose the potential role of a TLR4-MyD88-dependent pathway in the pathogenesis of acute anterior uveitis. The blockage of this pathway by anti-TLR4 may signal a new direction in the treatment of acute anterior uveitis.

## Introduction

Anterior uveitis is the most common form of uveitis. The etiology of uveitis is unclear, but it is speculated to be an autoimmune response resulting from a breakdown in the normal state of ocular immune privilege [1]. Extensive clinical and experimental evidence supports the role of a particular Gram-negative bacteria or its lipopolysaccharides (LPS) in the pathogenesis of noninfectious, immune-mediated acute anterior uveitis (AAU) [2]. Toll-like receptors (TLRs) are a family of pattern-recognition receptors of innate immunity that recognize unique molecular signatures of microbes, known as pathogen-associated molecular patterns (PAMPs) [3-5]. TLRs are the first line of host defense and TLR activation, by their respective PAMPs, result in proinflammatory cytokine cascades and the induction of both innate and adaptive immune responses. We have demonstrated a higher expression of TLR4 on uvea-resident tissue macrophages in endotoxin-induced acute anterior uveitis than is found in normal rats and we have proposed a pathogenic mechanism whereby LPS of Gram-negative bacteria (GNB) could initiate uveitis by activation of intraocular TLR4 and produce proinflammatory cytokines and chemokines for the recruitment of leukocytes to the eye [6]. McMenamin [7] reported that the uveal tract in mice, as in rats, contains rich networks of resident tissue macrophages. The networks of resident tissue macrophages in the uveal tract of mice closely resemble those in the peritoneal cavity. To further study the role of TLR4 on macrophages in acute anterior uveitis, we selected C3H/HeN mice (wild type) and C3H/HeJ mice (*TLR4* gene defect type), treated with an intraperitoneal injection of vibrio cholera endotoxin, to establish mice models with acute anterior uveitis. The anterior segment was observed with a slit lamp and analyzed with histopathologic examination. Expression of TLR4, myeloid differentiation factor 88 (MyD88), and nuclear factor kappa B (NF-κB), with or without LPS stimulation, was observed in isolated peritoneal macrophages from the C3H/HeN and the C3H/HeJ mice.

## Methods

### Animals

Adult male C3H/HeN mice (6–8 weeks old) were obtained from the Vital River Laboratory Animal Technology Co. Ltd (Beijing, China). Adult male C3H/HeJ mice (6–8 weeks old) were obtained from the Model Animal Research Center (Nanjing, China). All mice were housed in pathogen-free conditions in cycle of 12 h light/12 h dark with free access to food and water. The specimens included 60 mice; 30 were used for the in vivo experiment (n=5/per group). Of these specimens, 24 C3H/HeN mice and 18 C3H/HeJ mice were used for the in vitro experiment. Throughout this study, all procedures adhered to the Institute for Laboratory Animal Research guidelines (Guide for the Care and Use of Laboratory Animals).

### Experimental groups

Animals were randomly divided into six groups: control group, LPS groups (1 h, 3 h, 6 h, 12 h, and 24 h after the mice received an intraperitoneal injection of LPS, or 1 h, 3 h, 6 h, 12 h, and 24 h after mouse peritoneal macrophages were stimulated with LPS).

### Animal model

The mice received an intraperitoneal injection of 200 μg vibrio cholera (classical Biotype, serotype Ogawa, kindly provided by the Lanzhou Institute of Biologic Products Lanzhou, China) dissolved in 100 μl sterile saline (NS). The eyes were examined using a slit microscope before injection and after several different hours had elapsed.

### Histopathology

The mice were killed by an overdose of pentobarbital (100 mg/kg) after being immunized with LPS. The eyes of the mice were enucleated and placed in a 10% neutral buffered formalin solution for 24 h. After the stationary liquid was washed out, a tissue sample was immersed in 50%, 75%, 80%, 90%, and 100% alcohol for 1 h, respectively, to dehydrate. Next, the tissue was put into paraffin for 1 h×3 to embed it after being treated with xylene for 30 min. Sagittal sections (4 μm thick) were cut near the optic nerve head and stained with hematoxylin and eosin.

### Culture and LPS stimulation of peritoneal macrophages

The mice were injected, intraperitoneally, with 2 ml of 3% thioglycollate (Taigemei, Biotechnology, Beijing, China). After four days, peritoneal cells were collected by lavage with an average viability of 98%. The cell viability was evaluated using the trypan (Sigma, St. Louis, MO) blue exclusion test (0.4%). Cells were seeded onto 24-well plates (1×10^5^ cells/well) in RPMI 1640 medium (Hyclone, Logan, Utah), supplemented with 2 mM glutamine (Hyclone), antibiotics (100 U/ml of penicillin and 100 U/ml of streptomycin), and 10% heat-inactivated fetal bovine serum (Hyclone) for 24 h to allow the macrophages to adhere to the plates. Nonadherent cells were subsequently removed by washing with Hank's balanced salt (HBSS) solution, confirmed with F4/80 stain. The adherent macrophages were grown in pre-placed coverslips in RPMI 1640 medium, containing 10% fetal bovine serum, and antibiotics. Macrophages, in the presence or absence of LPS, were used for the experiments. The anti-TLR4 monoclonal antibody (rat monoclonal antibody; Santa Cruz Biotechnology, Santa Cruz, CA) group, with adherent macrophages, was pretreated with anti-TLR4 monoclonal antibody (with a final concentration of 10 μg/ml) for 1 h, then washed, three times, with HBSS solution. Subsequent, identical steps were taken with the other groups.

### Immunofluorescence

The adherent cells were washed with PBS, fixed in freshly prepared 4% paraformaldehyde in PBS for 15 min at room temperature, washed, three-times, with PBS, permeabilized with HEPES-Triton buffer (20 mM HEPES, 300 mM sucrose, 50 mM NaCl, 3 mM MgCl_2_, 0.5% Triton X-100, pH 7.4) on crushed ice for 1 h, and then washed, three times, with PBS. The cells were blocked with PBS containing 10% BSA for 1 h, at room temperature, and incubated with F4/80 (rat monoclonal antibody; Santa Cruz Biotechnology), TLR4, MyD88 (rabbit polyclonal antibody; Santa Cruz Biotechnology), and NF-κB (mouse monoclonal antibody; Santa Cruz Biotechnology), respectively, in a humidified chamber, at 4 °C overnight. (all antibodies 1:50 in 10% BSA/PBS). Excessive antibodies were removed by washing the coverslips, three times, with PBS. The cells were incubated with fluorescein-conjugated goat anti-rabbit IgG, rhodamine-conjugated goat anti-rat IgG, and goat anti-mouse IgG (1:200 in PBS; Zhongshan Goldbridge Biotechnology, Beijing, China) for 2 h and were protected from light and room temperature. After being washed, three times, with PBS, the cells were mounted onto a glass slide, using a mounting medium. Negative controls included replacing the first or second primary antibody with species- and isotype-matched irrelevant antibodies. Blank controls included replacing the first or second primary antibody with PBS. Slides were examined under a fluorescence microscope (Leica-DM-4000B; Leica, Wetzlar, Germany). Five high power fields were selected to analyze each stain by a single masked observer. Images were captured using an inverted confocal laser-scanning microscope (Leica-DM-IRE2; Leica).

### Data processing and statistical analysis

Leica QWin software was used to analyze the intensity of the fluorescence of the cell area. Statistic analysis was performed using SPSS17.0 (SPSS Inc., Chicago, IL) software. For multiple comparisons, different groups were analyzed using the one-way ANOVA technique, followed by Fisher’s Least Significant Difference Procedure (LSD) tests. A p-value, less than or equal to 0.05, was considered significant.

## Results

### Clinical manifestation of EIU

**Figure 1:**
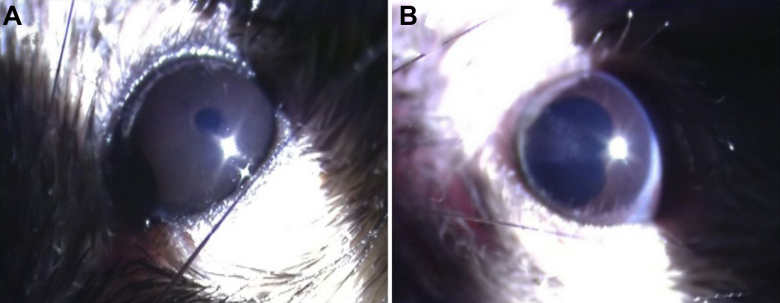
The clinical manifestation of C3H /HeJ and C3H /HeN mice after injection of endotoxin. **A**: No anterior segment inflammation in C3H /HeJ mice at 24 h after injection of endotoxin. **B**: Posterior synechia, kidney shaped pupil after mydriasis with compound tropicamide in C3H /HeN mice at 24 h after injection of endotoxin.

No anterior segment inflammation was observed in the C3H/HeJ mice after LPS injection (Figure 1A). Ocular inflammatory response was detected in the C3H/HeN mice after LPS injection and was consistent with manifestation of AAU. Twenty-four hours after injection, the pupil was irregular and posterior synechia could be seen after mydriasis (Figure 1B).

### Histologic Changes

**Figure 2:**
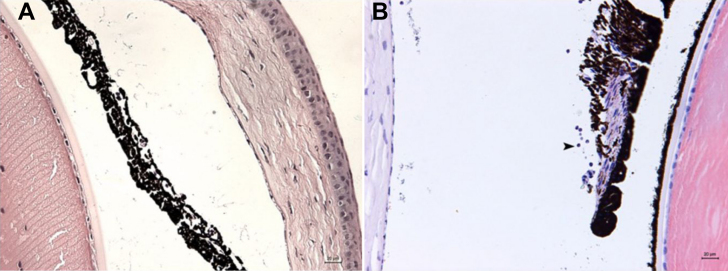
HE staining in C3H /HeN mice at 24 h after injection of endotoxin. **A**: No inflammatory cells were observed in the anterior chamber of C3H /HeJ mice (bar=20 μm). **B**: Thickness of iris stroma layer and a large number of neutrophilic granulocytes were observed in C3H /HeN mice (Arrow indicated positive cells, bar=20 μm).

HE staining results were consistent with clinical manifestations in the wild-type and gene-deficient mice after LPS immunization. No inflammatory cells were detected in the anterior chamber of the C3H/HeJ mice in HE staining (Figure 2A), but infiltration of inflammatory cells and fibrin exudations could be seen in the anterior and posterior chamber of the C3H/HeN mice. The blood vessels thickened in the dilated irises and in the iris stroma. The majority of inflammatory cell infiltration was detected in the iris-ciliary body (Figure 2B).

### Cell Identification

**Figure 3:**
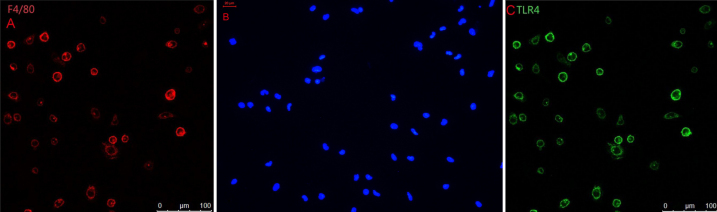
Immunohistochemical studies for TLR4 and F4/80. **A**: Unstimulated C3H/HeN mouse peritoneal macrophages were marked with F4/80 staining. Cells were approximately round. **B**: No staining was seen when under identical experimental conditions when the primary antibody was replaced with normal IgG at the same concentration (negative control). **C**: The TLR4+ cells of C3H/HeN mice possessed round-ovoid morphology, expressed in the membrane without LPS stimulation.

Unstimulated mouse peritoneal macrophages were marked with F4/80 staining. Cells were approximately round (Figure 3A). The nucleus of the cells was round, kidney-shaped, or irregular. F4/80 and TLR4 could not be detected in the negative group (Figure 3B).

### Fluorescence intensity of TLR4 and cell morphology with or without LPS stimulation

**Figure 4:**
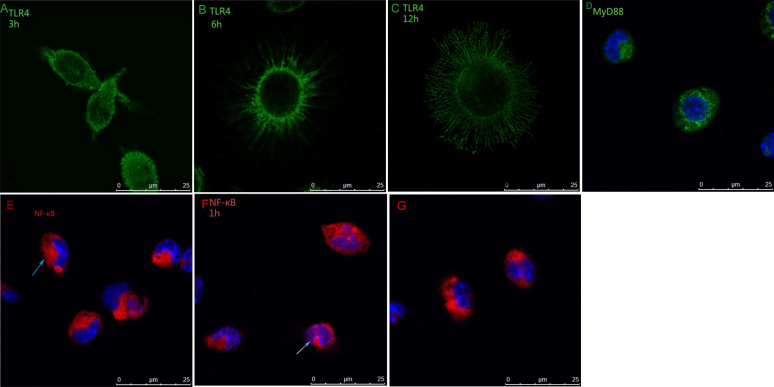
The shape changed after LPS stimulation. MyD88 and NF-κB were expressed differently before and after LPS stimulation of C3H/HeN mouse peritoneal macrophages. **A**: Three hours after LPS stimulation, the cell elongated significantly. **B**: Six hours after LPS stimulation, the cell showed extended pseudopodia and protrusions. **C**: Twelve hours after LPS stimulation, the cell extended a large number of pseudopodia and protrusions and the size of cell reached its peak. **D**: In unstimulated C3H/HeN mouse peritoneal macrophages without LPS stimulation, MyD88 were expressed in the cytoplasm and nuclei. **E**: In C3H/HeN mouse peritoneal macrophages without LPS stimulation, NF-κB was expressed in the cytoplasm. **F**: One hour after LPS stimulation of C3H/HeN mouse peritoneal macrophages, NF-κB were expressed in the nuclei. **G**: NF-κB was expressed in the cytoplasm after the blockage of TLR4 with MTS510.

**Figure 5:**
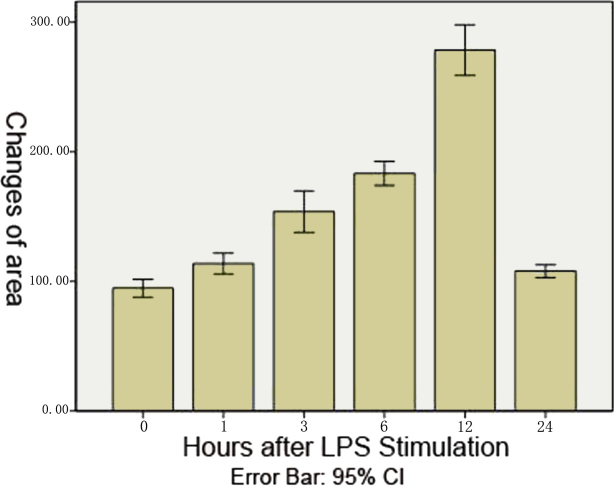
Changes of area of C3H/HeN mouse peritoneal macrophages after LPS stimulation. The shape of C3H/HeN mouse peritoneal macrophages changed after LPS stimulation, primarily with regard to the enlargement of cells. Twelve hours after LPS stimulation, cell size reached its peak, and then gradually became smaller.

In the unstimulated C3H/HeN mouse peritoneal macrophages, TLR4 was expressed on the membrane (Figure 3C). The fluorescence intensity of TLR4 in C3H/HeN mouse peritoneal macrophages was stronger than in the C3H/HeJ mouse macrophanges (F=314.007, p<0.0001), but no significant difference (F=1.642, p=0.155) was noted in the areas of different mouse macrophanges. The shape of the C3H/HeN mouse peritoneal macrophages changed after LPS stimulation, including the enlargement of cells, extended pseudopodia, and protrusions (Figure 4A-C). Twelve hours after LPS stimulation, cell size reached its peak, and then gradually became smaller (Figure 5). The cell areas showed a statistically significant difference among all the groups (F=216.369, p<0.0001). The results of the Least Significant Difference Procedure (LSD) tests, used for multiple comparisons, showed no significant difference between the control group and the 24 h group (p=0.055); however, for the rest of the groups, statistically significant difference was shown between every two groups (p<0.0001). There was no significant difference (p=0.25) in the size of the peritoneal macrophages of the different groups of C3H/HeJ mice after LPS stimulation.

### Immunofluorescence of MyD88 and NF-κB

**Figure 6:**
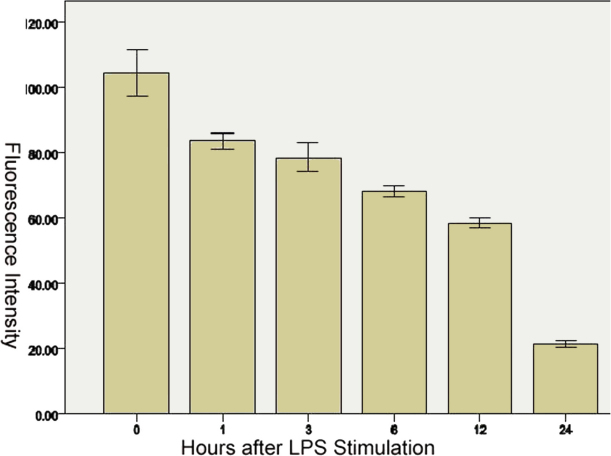
Change of fluorescein intensity of MyD88 in C3H/HeN mice peritoneal macrophages after stimulation with LPS. The fluorescence intensity gradually decreased with passing time.

**Figure 7:**
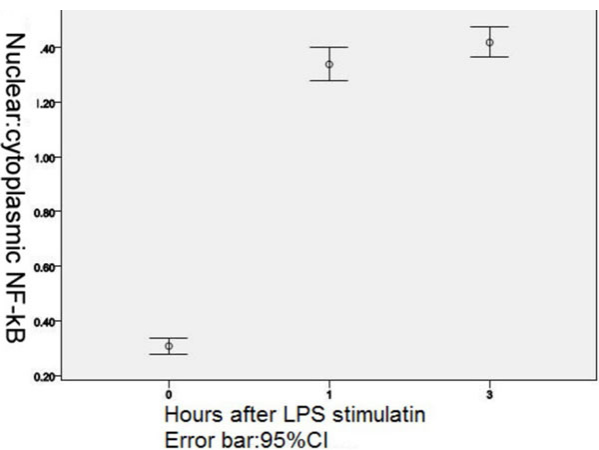
Nuclear factor translocation with LPS stimulation in C3H/HeN mouse peritoneal macrophages. Immunofluorescence staining of nuclear/cytoplasmic ratios of NF-кB nuclear translocation in LPS stimulated macrophages.

In unstimulated C3H/HeN and C3H/HeJ mouse peritoneal macrophages, MyD88 was expressed in the cytoplasm and nuclei (Figure 4D). There was no significant difference in fluorescence intensity between the two types of mice (p=0.315). After LPS stimulation, the fluorescence intensity gradually decreased with the time past (Figure 6), a statistically significant difference was observed in MyD88 fluorescence intensity in C3H/HeN mouse peritoneal macrophages among all groups (F=393.485, p<0.0001).There was statistically significant difference between every two groups when the LSD tests were used for multiple comparisons (comparison between 1 h and 3 h, p=0.017; other groups, p<0.0001). In all the groups of C3H/HeJ mouse peritoneal macrophages, with LPS stimulation, there was no significant difference (p=0.421) in MyD88 fluorescence intensity. In C3H/HeN and C3H/HeJ mouse peritoneal macrophages, without LPS stimulation, NF-κB was expressed in the cytoplasm (Figure 4E). One hour after LPS stimulation, NF-κB was expressed in the nuclei (Figure 4F), and the nuclear/cytoplasmic ratio gradually increased until 3 h after LPS stimulation (Figure 7), but 6 h after LPS stimulation, the expression of NF-κB in C3H/HeN mouse peritoneal macrophages could not be detected. NF-κB was detected in the membrane in C3H/HeJ mouse peritoneal macrophages 1 h after LPS stimulation, and it was still expressed in the membrane until 24 h after LPS stimulation.

### The expression of TLR4, MyD88 and NF-κB in the Anti-TLR4 monoclone antibody MTS510 group

The expression of TLR4, MyD88, and NF-κB, in the C3H/HeN mouse peritoneal macrophages first incubated with MTS510 for 1 h and then stimulated with LPS, were the same as the expression of C3H/HeJ mouse peritoneal macrophages stimulated with LPS; however, the expression of NF-κB in the cytoplasmic and nuclear translocation could not be detected (Figure 4G).

## Discussion

Uveitis is a common inflammatory disease that is a potential threat to visual loss. It primarily affects the iris, the ciliary body, and the choroid [8]. At present, the pathogenic mechanisms of uveitis is unclear. The majority of uveitis may be caused by noninfectious factors. Only a small part of the infectious uveitis is due to pathogen invasion. Acute anterior uveitis, especially HLA-B27-associated AAU, is a common noninfectious uveitis, but clinical and laboratory research have proven that gram-negative bacteria species, such as *Klebsiella*, *Salmonella*, *Yersinia*, and *Shigella*, can trigger it [2].

The variation of expression of TLR4, and its downstream signal transduction molecules MyD88 and NF-κB after LPS stimulation in vitro, presumed the potential role of a TLR4-MyD88 dependent pathway in the pathogenesis of acute anterior uveitis. Up on LPS recognition, TLR4 undergoes oligomerization and recruit its downstream adaptors through interactions with the TIR domains. The TIR domain of TLR4 is critical for signal transduction. MyD88 is one of the TIR domain-containing adaptor protein, which contains a death domain, can recruit other death domain containing molecules through homotypic interactions [9]. In our study, we found MyD88 decreased with times after LPS stimulation. We suggest it was consumed by the reaction.After MyD88 activation, another adaptor protein, TRAF6 (TNF receptor-associated factor 6), is critical for the MyD88-dependent pathway. It leads to the phosphorylation of IκB proteins, which leads to NF-κB/I-κB trimer complex degradation. Subsequently, NF-κB is activated and transferred into the nucleus [9]. Noursadeghi [10] reported that measurement of nuclear fluorescence alone does not distinguish NF-κB nuclear translocation from increased background levels of NF-κB expression or artifactual differences in staining intensity. Therefore, nuclear and cytoplasmic staining intensities were compared to indicate the nuclear/cytoplasmic ratio as a relative measure of nuclear localization. Therefore, we measured the nuclear/cytoplasmic ratio to show the nuclear translocation of NF-κB. In our study, we found that C3H/HeN mice could be induced with EIU, but C3H/HeJ mice could not be induced with EIU, the same as Li et al. [11] reported. Cell culture revealed that, with LPS stimulation, C3H/HeN mouse macrophage could be activated, cell size was increased, and NF-κB was translocated into the nucleus. This may be an important reason for iris congestion, anterior chamber flare, and clinical manifestation.

Our previous study revealed that in endotoxin-induced uveitis rat model macrophages, with shape alterations, were only located in the stroma bordering the iris endothelial layer [6]. In cell cultures, in vitro stimulated with LPS, we found that the cells located in the stroma were optimally positioned to access and respond to the LPS of invasive organisms breaking the blood–ocular barrier. Activation of TLR4 on macrophages, using LPS stimulation, resulted in the activation of the transcriptional factor and nuclear factor-κB (NF-κB), via an immunostimulatory intracellular signaling pathway.

LPS stimulation of mammalian cells occurs through a series of interactions with several proteins, including the LPS binding protein (LBP), CD14, MD-2, and TLR4 [12,13]. LBP is a soluble shuttle protein that directly binds to LPS and facilitates the association between LPS and CD14 [14,15]. CD14 is a glycosylphosphatidylinositol-anchored protein that also exists in a soluble form. CD14 facilitates the transfer of LPS to the TLR4/MD-2 receptor complex and modulates LPS recognition [16]. MD-2 is a soluble protein that non-covalently associates with TLR4, but it can directly form a complex with LPS in the absence TLR4 [17-19].

TLR4 is essential for LPS signaling; however, overexpression of TLR4 does not confer LPS responsiveness. MD-2, a small protein that lacks a transmembrane domain, is identified to associate with the extracellular domain of TLR4. Importantly, expression of MD-2 confers a responsiveness to LPS. Studies have shown that cells, transfected with TLR4 alone, were unresponsive to LPS, but cells transfected with TLR4 and MD2 were strongly activated strongly [20,21].

MTS 510, an anti-mouse TLR4 mAb, is reported to block LPS-induced NF-κB activation [22]. Akashi et al. [22] cloned mouse MD-2 molecularly and established a unique mAb MTS510 which reacted selectively with mouse TLR4-MD-2, but not with TLR4 alone. In our study, NF-κB nuclear translocation could not be detected in C3H/HeN mouse macrophages, pre-cultured with MTS510 for 1 h and then stimulated by LPS. We believed that the macrophage was unresponsive to LPS at that time.

How does MTS510 affect LPS-induced NF-κB activation? We presume it can be attributed to either the disrupted association of TLR4 and MD-2, shedding from the cell surface, or to internalization. Interestingly, C3H/HeJ mice, which have a mutant *Lps* allele (*Lps^d/d^*) [23], confer hyporesponsiveness to LPS. Poltorak [24] compared C3H/HeN and C3H/HeJ mice and revealed that the point mutation, in latter q32–33, resulted in the proline being replaced by histidine, and the proline was a key component of TLR4 signaling; however, C3H/HeJ mice could express TLR4. Here we discovered that C3H/HeJ mouse macrophages could express TLR4, MyD88 after LPS stimulation, but the NF-κB was translocated from the cytoplasm to the membrane. The signal could not be transferred, and the macrophage had no response to LPS. We thought that, because of gene defection, the signals might change, then the IκB complex, which combined with NF-κB to make an inactive state in the cytoplasm, might be stimulated by an incorrect signal, resulting in the expression of NF-κB in the membrane. As the signal could not be conducted, the macrophages could not response to LPS stimulation.

### Conclusions

In summary, the present study presumed that TLR4 activated its downstream signaling molecules through a MyD88-dependent pathway and may play an important role in the pathogenesis of AAU. The blockage of this pathway by anti-TLR4 may result in a new direction for the treatment of acute anterior uveitis.

## References

[1] Chang JH Hampartzoumian T Everett B Lloyd A McCluskey PJ Wakefield D Changes in toll-like receptor (TLR)-2 and TLR4 expression and function but not polymorphisms are associated with acute anterior uveitis. Invest Ophthalmol Vis Sci 2007 48 1711 7 17389503 10.1167/iovs.06-0807

[2] Chang JH McCluskey PJ Wakefield D Acute anterior uveitis and HLA-B27. Surv Ophthalmol 2005 50 364 88 15967191 10.1016/j.survophthal.2005.04.003

[3] Chang JH McCluskey PJ Wakefield D Toll-like receptors in ocular immunity and the immunopathogenesis of inflammatory eye disease. Br J Ophthalmol 2006 90 103 8 16361678 10.1136/bjo.2005.072686 PMC1856909

[4] Takeda K Kaisho T Akira S Toll-like receptors. Annu Rev Immunol 2003 21 335 76 12524386 10.1146/annurev.immunol.21.120601.141126

[5] Takeda K Akira S Toll-like receptors in innate immunity. Int Immunol 2005 17 1 14 15585605 10.1093/intimm/dxh186

[6] Chen W Hu X Zhao L Li S Lu H Expression of toll-like receptor 4 in uvea-resident tissue macrophages during endotoxin-induced uveitis. Mol Vis 2009 15 619 28 19347047 PMC2664840

[7] McMenamin PG Dendritic cells and macrophages in the uveal tract of the normal mouse eye. Br J Ophthalmol 1999 83 598 604 10216062 10.1136/bjo.83.5.598 PMC1723050

[8] Munoz-Fernandez S Martın-Mola E Uveitis. Best Pract Res Clin Rheumatol 2006 20 487 505 16777578 10.1016/j.berh.2006.03.008

[9] Lu YC Yeh WC Ohashi PS LPS/TLR4 signal transduction pathway. Cytokine 2008 42 2I 145 51 18304834 10.1016/j.cyto.2008.01.006

[10] Noursadeghi M Tsang J Haustein T Miller RF Chain BM Katz DR Quantitative imaging assay for NF-κB nuclear translocation in primary human macrophages. J Immunol Methods 2008 329 194 200 18036607 10.1016/j.jim.2007.10.015 PMC2225449

[11] Li Q Peng B Whitcup SM Jang SU Chan CC Endotoxin induced uveitis in the mouse:susceptibility and genetic controls. Exp Eye Res 1995 61 629 32 8654505 10.1016/s0014-4835(05)80056-9

[12] Gioannini TL Weiss JP Regulation of interactions of Gram-negative bacterial endotoxins with mammalian cells. Immunol Res 2007 39 249 60 17917069 10.1007/s12026-007-0069-0

[13] Miyake K Innate immune sensing of pathogens and danger signals by cell surface Toll-like receptors. Semin Immunol 2007 19 3 10 17275324 10.1016/j.smim.2006.12.002

[14] Tobias PS Soldau K Ulevitch RJ Isolation of a lipopolysaccharide binding acute phase reactant from rabbit serum. J Exp Med 1986 164 777 93 2427635 10.1084/jem.164.3.777 PMC2188379

[15] Wright SD Tobias PS Ulevitch RJ Ramos RA Lipopolysaccharide (LPS) binding protein opsonizes LPS-bearing particles for recognition by a novel receptor on macrophages. J Exp Med 1989 170 1231 41 2477488 10.1084/jem.170.4.1231 PMC2189482

[16] Wright SD Ramos RA Tobias PS Ulevitch RJ Mathison JC CD14, a receptor for complexes of lipopolysaccharide (LPS) and LPS binding protein. Science 1990 249 1431 3 1698311 10.1126/science.1698311

[17] Shimazu R Akashi S Ogata H Nagai Y Fukudome K Miyake K Kimoto M MD-2, a molecule that confers lipopolysaccharide responsiveness on Toll-like receptor 4. J Exp Med 1999 189 1777 82 10359581 10.1084/jem.189.11.1777 PMC2193086

[18] Nagai Y Akashi S Nagafuku M Ogata M Iwakura Y Akira S Kitamura T Kosugi A Kimoto M Miyake K Essential role of MD-2 in LPS responsiveness and TLR4 distribution. Nat Immunol 2002 3 667 72 12055629 10.1038/ni809

[19] Gioannini TL Teghanemt A Zhang D Coussens NP Dockstader W Ramaswamy S Weiss JP Isolation of an endotoxin-MD-2 complex that produces Toll-like receptor 4-dependent cell activation at picomolar concentrations. Proc Natl Acad Sci USA 2004 101 4186 91 15010525 10.1073/pnas.0306906101 PMC384716

[20] Abreu MT Vora P Faure E Thomas LS Arnold ET Arditi M Decreased expression of toll-Like receptor-4 and MD-2 correlates with intestinal epithelial cell protection against dysregulated proinflammatory gene expression in response to bacterial lipopolysaccharide. J Immunol 2001 167 1609 16 11466383 10.4049/jimmunol.167.3.1609

[21] Cario E Toll-like receptors and intestinal defense: Molecular basis and therapeutic implications. Expert Rev Mol Med 2003 5 1 15 14585165 10.1017/S1462399403006501

[22] Akashi S Shimazu R Ogata H Nagai Y Takeda K Kimoto M Cell surface expression and lipopolysaccharide signaling via the toll-like receptor 4-MD-2 complex on mouse peritoneal macrophages. J Immunol 2000 164 3471 5 10725698 10.4049/jimmunol.164.7.3471

[23] Li Q Peng B Whitcup SM Jang SU Chan CC Endotoxin induced uveitis in the mouse: Susceptibility and genetic control. Exp Eye Res 1995 61 629 32 8654505 10.1016/s0014-4835(05)80056-9

[24] Poltorak A Smirnova I He X Liu MY Van Huffel C McNally O Birdwell D Alejos E Silva M Du X Thompson P Chan EK Ledesma J Roe B Clifton S Vogel SN Beutler B Genetic and physical mapping of the LPS locus: Identification of the toll-4 receptor as a candidate gene in the critical region. Blood Cells Mol Dis 1998 24 340 55 10087992 10.1006/bcmd.1998.0201

